# The Nurse's Lack of Business Habits

**Published:** 1919-12-20

**Authors:** 


### THE MATRONS' AND SISTERS' DEPARTMENT.

### THE NURSE'S LACK OF BUSINESS HABITS.

MORE than once in these columns attention has been drawn to defects in nurses which point to deficiency in their training. The usual reply of those responsible for the curriculum in training-schools is that the business of the hospital is to teach the probationer to become a nurse, and that beyond those limits they do not propose to trespass. But there are certain omissions in the equipment of the nurse which though they are not discernible as defects from a strictly nursing point of view are yet plainly responsible for the failure of nurses in various spheres to which nursing introduces them. By far the most fatal defect from the point of view of the nurse herself is the lack of business habits.

It may be at once granted that the nurse may perform to admiration all her duties in the sick-room without having any notion of what is ordinarily called business habits. But she is singularly at a loss in all that lies outside that limited area if she has not acquired some knowledge of how to regulate her income, some clear notion of the value of money, of the cost of the things she uses, of the best methods of planning out her salary, and of investing the surplus, of conducting her correspondence, of applying for vacant posts, of understanding a contract, of filling in business forms, of writing reports. Is it reasonable to expect her to come fully equipped in these particulars when she first enters the hospital? A moment's reflection will show that this kind of preparation is impossible for the probationer, because in becoming a nurse she enters upon entirely different obligations from any she has hitherto incurred. Home life is not, and cannot be, an adequate business preparation for the peculiar financial position in which the private nurse is placed. Still less can it prepare women for the exactitude needed by the public health nurse, or for the book-keeping so often required of matrons in little hospitals, or guard her from the temptations of lavish expenditure in a career exposed to such violent fluctuations of income as that of nursing. Unless she receive counsel and instruction during her time of training in the art of surmounting business difficulties she will run great risks, and may easily become the prey of those kind individuals who are ever on the look-out to dip their fingers into the pockets of the unbusinesslike. In other words she will prove a failure, and will swell the ranks of those who have to be rescued from the consequences of their ignorance.

The need is for a practical course of business preparation for their career, adapted to the needs of nurses in general and private nurses in particular. There are, doubtless, manuals of a helpful character which could be utilised in framing such a course, but these commonly furnish a great deal of information not strictly required by the nurse, and omit many of the points on which she chiefly requires guidance. Practical talks given by a sister of wide experience who is acquainted with the pitfalls which lurk in the nurse's career would doubtless have more effect than printed matter, but practice in some of the directions we have already enumerated is indispensable. This might come in as part of the ordinary class system, and take the form of useful exercises for the probationer to work out. For instance, the whole co-operative system of private nursing needs explaining. Lack of mastery of its essential principles is, at the present moment, imperilling the future of the oldest co-operation formed among nurses. Ignorance has paved the way for reckless and insincere agitation.

The business principle ought to begin with the probationer's own business place in the hospital. She ought to know clearly the exact cost of the board, house-room, laundry, uniform and education which make part of her salary. Always she should be led to see her position as part of a business concern. The exact value in money of her emoluments should be set forth on the paper which she signs at the outset, and should be kept ever in view. Were this done we should hear no more nonsense about nursing at ½d. an hour. The value of her board and lodging as a private, district, or institution nurse, ought to be as clear to her mind as the amount of money she gets every month. Help should be given her in forecasting her future. The insurance tables of the Royal National Pension Fund for Nurses should be the basis of calculations made by herself in estimating what measure of independence she may secure and on what salary. She should be taught simple book-keeping and how to keep personal accounts of her income and expenditure. She should also receive instruction in the details of keeping a banking account.

The first step to reform is to discard the old-fashioned idea that the inculcation of business habits is no legitimate part of a nurse's training.

